# A genetic variant in SLC28A3, rs56350726, is associated with progression to castration-resistant prostate cancer in a Korean population with metastatic prostate cancer

**DOI:** 10.18632/oncotarget.18298

**Published:** 2017-05-30

**Authors:** Jung Ku Jo, Jong Jin Oh, Yong Tae Kim, Hong Sang Moon, Hong Yong Choi, Seunghyun Park, Jin-Nyoung Ho, Sungroh Yoon, Hae Young Park, Seok-Soo Byun

**Affiliations:** ^1^ Department of Urology, Hanyang University Hospital, Seoul, Korea; ^2^ Department of Urology, Seoul National University College of Medicine, Seoul National University Bundang Hospital, Seongnam, Korea; ^3^ Department of Urology, Hanyang University Guri Hospital, Guri-si, Korea; ^4^ Department of Electrical and Computer Engineering, Seoul National University, Seoul, Korea; ^5^ Biomedical Research Institute, Seoul National University Bundang Hospital, Seongnam, Korea

**Keywords:** metastasis, prostate cancer, castration, genetic variation

## Abstract

**Background:**

Genetic variation which related with progression to castration-resistant prostate cancer (CRPC) during androgen-deprivation therapy (ADT) has not been elucidated in patients with metastatic prostate cancer (mPCa). Therefore, we assessed the association between genetic variats in mPCa and progession to CRPC.

**Results:**

Analysis of exome genotypes revealed that 42 SNPs were significantly associated with mPCa. The top five polymorphisms were statistically significantly associated with metastatic disease. In addition, one of these SNPs, rs56350726, was significantly associated with time to CRPC in Kaplan-Meier analysis (Log-rank test, *p* = 0.011). In multivariable Cox regression, rs56350726 was strongly associated with progression to CRPC (HR = 4.172 95% CI = 1.223-14.239, *p* = 0.023).

**Materials and Methods:**

We assessed genetic variation among 1000 patients with PCa with or without metastasis, using 242,221 single nucleotide polymorphisms (SNPs) on the custom HumanExome BeadChip v1.0 (Illuminam Inc.). We analyzed the time to CRPC in 110 of the 1000 patients who were treated with ADT. Genetic data were analyzed using unconditional logistic regression and odds ratios calculated as estimates of relative risk of metastasis. We identified SNPs associated with metastasis and analyzed the relationship between these SNPs and time to CRPC in mPCa.

**Conclusions:**

Based on a genetic variation, the five top SNPs were observed to associate with mPCa. And one (SLC28A3, rs56350726) of five SNP was found the association with the progression to CRPC in patients with mPCa.

## INTRODUCTION

Prostate cancer (PCa) is the most commonly diagnosed cancer in men, and the second most frequent cause of cancer-related death among them, in the USA [1]. Moreover, PCa is the second most common newly diagnosed cancer worldwide [2]. The presence or absence of metastasis is an important factor in PCa and patients presenting with metastases at diagnosis demonstrate worse survival rates. Therefore, appropriately timed interventions, based on early diagnosis, can lead to improved clinical outcomes for PCa patients. Novel biomarkers have the potential to facilitate early intervention in PCa.

In the era of next-generation sequencing (NGS), the understanding of the genetic basis of PCa has improved [3]. Berger et al. performed the first whole genome sequencing analysis of human PCa [4]. Their results indicate that genomic rearrangements, including chromosomal aberrations, might be a major mechanism driving prostate carcinogenesis. However, they found that point mutations in PCa are relatively rare compared to chromosomal rearrangements. However, the mechanisms underlying metastatic spread in PCa remain unclear. Deeper understanding of the mechanisms of metastasis is necessary for optimization of the efficacy both of approved and novel agents against metastases in PCa and is key for the design of personalized medicine in patients with metastatic PCa (mPCa).

Androgen-deprivation therapy (ADT) is the standard treatment for advanced or recurrent PCa. The initial response to ADT is good in the majority of PCa cases and most PCa is androgen-dependent. However, eventual recurrence of PCa occurs during ADT in the majority of cases, and is defined as castration-resistant prostate cancer (CRPC). Various mechanisms have been suggested for the development of the castration-resistant state, including mutations and amplification of the AR gene, ligand-independent AR activation, and dysregulation of the AR gene [5, 6]. Moreover, intratumoral PCa cells produce androgens and have a key role in the sustenance of CRPC. Patients with metastatic CRPC have worse outcomes than those with CRPC but without metastasis. At present, little is known, including regarding the influence of genetic variants, about either metastatic PCa or progression to CRPC. Moreover, genetic variation which related with progression to CRPC during ADT has not been elucidated in patients with mPCa.

In this study, we aimed to analyze the association of genetic variants with mPCa in Korean men. Furthermore, we hypothesized that SNPs associated with mPCa could also be related to time to progression to CRPC. Therefore, we analyzed the relationship between SNPs associated with mPCa and time to progression to CRPC.

## RESULTS

The clinical characteristics of the total study population are summarized in Table 1. The median age was 68 (IQR: 63–72) years, the median BMI was 24.3 (IQR: 22.6–25.9) kg/m2, the median PSA was 8.8 (IQR: 5.6–17.4) ng/ml, and the median prostate volume was 33.4 (IQR: 27–43.6) cc. Of the total of 1000 patients included in our study, 41 (4.1%) patients presented with metastatic PCa. The median follow-up period among the 110 patients who were treated with hormone therapy was 39 months (IQR: 18–61). Twenty nine (26%) of the 110 ADT-treated patients demonstrated progression to CRPC. The median follow-up period for the 29 patients who progressed to CRPC was 39 months (IQR: 15.5–68). Other clinical characteristics of the patients treated with hormone therapy are presented in Table 2. We analyzed the nadir testosterone level according to progression to CRPC in patients with ADT. It was not observed significant difference between two groups (*p* = 0.455).

**Table 1:** Characteristics of patients according to metastasis

Variables	No metastasis	Metastasis	*p*-value
*N*	959	41	
Age, yr			
Mean (SD)	67.3 ± 7.3	71.1 ± 6.9	0.001
Median (IQR)	68 (10)	74 (12)	
BMI, kg/m2			
Mean (SD)	24.5 ± 8.3	23.3 ± 3.4	0.399
Median (IQR)	24.3 (3.3)	23.0 (5.0)	
PSA			
Mean (SD)	17 ± 33.7	370.6 ± 581.3	0.11
Median (IQR)	8.5 (11)	60.7 (44.7–114.2)	
Prostatic volume			
Mean (SD)	37.4 ± 17.3	42.5 ± 18.3	0.647
Median (IQR)	33.3 (16.1)	40.4 (18.4)	
Gleason score, n (%)			
≤ 6	377 (40.5)	0	< 0.001†
7	392 (42.1)	4 (11.8)	
≥ 8	163 (17.5)	34 (88.2)	
Clinical stage, *n* (%)			
T1	565 (58.9)	1 (2.4)	< 0.001†
T2	291 (30.3)	6 (14.6)	
T3	88 (9.2)	27 (65.9)	
T4	2 (0.2)	7 (17.1)	
Treatment, *n* (%)			
Radical prostatectomy	836 (87.2)	0	< 0.001†
Active surveillance	23 (2.4)	0	
HTx	70 (7.3)	39 (95.1)	
Radition therapy	8 (0.8)	0	
SNPs, *n*			
rs2241714	714	16	< 0.001
rs143790069	33	9	< 0.001
rs72821581	26	7	< 0.001
rs75992542	47	8	< 0.001
rs56350726	147	17	< 0.001

Student *t* test, † chi-square test, BMI = body mass index, PSA = prostate specific antigen, HTx = hormone therapy

**Table 2:** Characteristics of patients according to the progression to CRPC

Variables	No progression group	Progression group	*p*-value
*N*	74	26	
Age, yr			
Mean (SD)	75.9 ± 7.4	71.3 ± 5.7	0.005
Median (IQR)	78 (7)	74 (8)	
BMI, kg/m2			
Mean (SD)	23.7 ± 3.2	22.9 ± 3.4	0.349
Median (IQR)	23.6 (4.3)	21.7 (5.4)	
PSA			
Mean (SD)	122.2 ± 302.7	335.5 ± 584.2	0.085
Median (IQR)	20.3 (41)	60.7 (32.5–105.4)	
Prostatic volume			
Mean (SD)	40.8 ± 22.7	44.8 ± 27.3	0.525
Median (IQR)	34.3 (26.5)	45.5 (24)	
Gleason score, *n* (%)			
≤ 6	15 (17.4)	0	< 0.001
7	39 (45.3)	4 (10.5)	
≥ 8	32 (37.2)	34 (89.5)	
Clinical stage, *n* (%)			
T1	19 (25.7)	1 (3.8)	0.01
T2	23 (31.1)	5 (19.2)	
T3	28 (37.8)	15 (57.7)	
T4	4 (5.4)	5 (19.2)	
SNPs, *n*			
rs2241714	53	14	0.193
rs143790069	8	5	0.231
rs72821581	5	3	0.395
rs75992542	10	5	0.42
rs56350726	13	8	0.121

Student t test, † chi-square test, BMI = body mass index, PSA = prostate specific antigen

We consider time to nadir PSA and nadir PSA level are more important to clinical relevant impact. Therefore, we analyzed these variables according to progression to CRPC. Time to nadir PSA level was not significant difference according to progression to CRPC (*p* = 0.063). However, nadir PSA level was observed significant difference according to progression to CRPC (*p* < 0.001).

**Figure 1:**
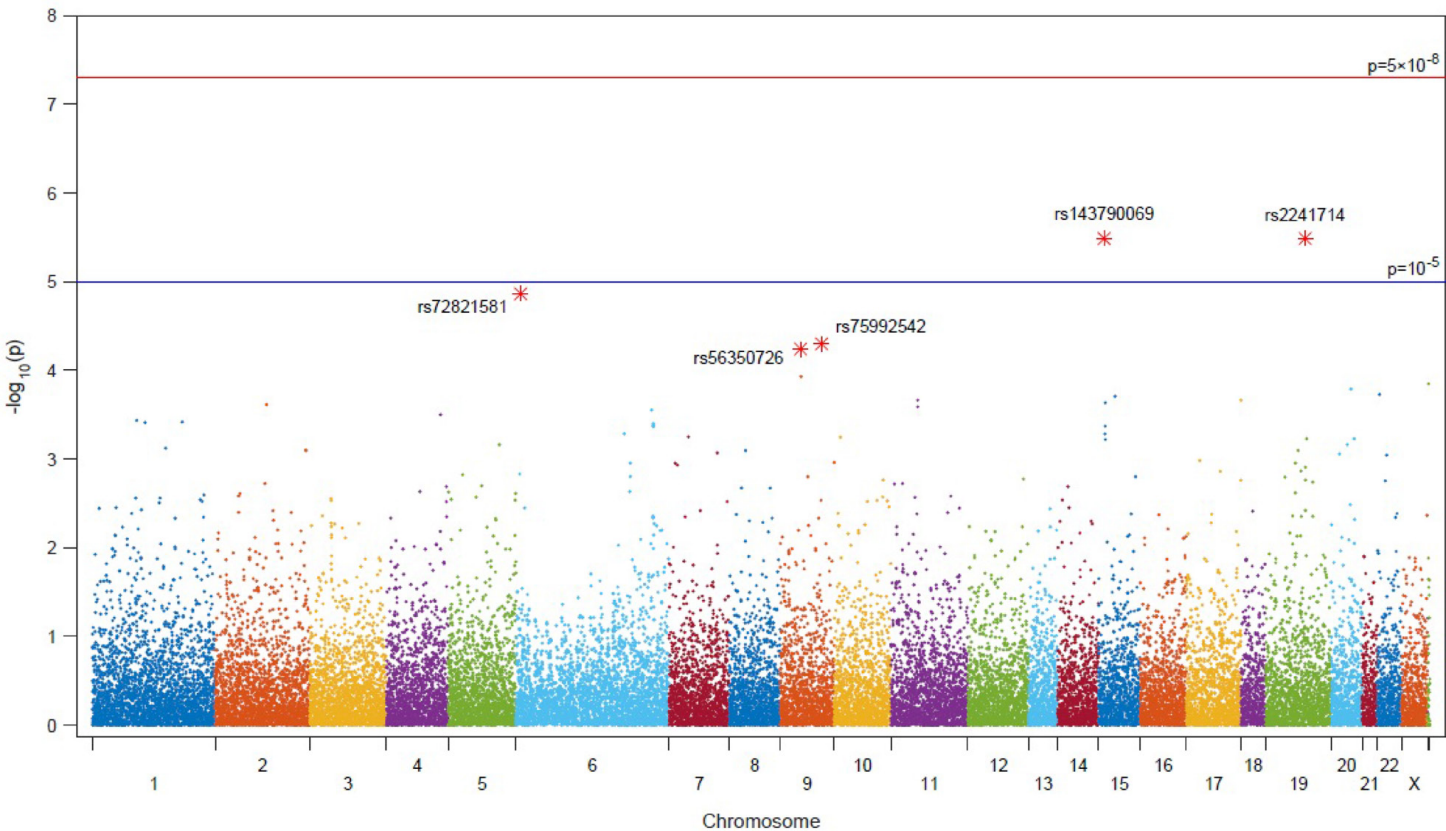
Manhattan plot of associations with metastatic PCa from analysis of 242,186 single-nucleotide polymorphisms on a custom HumanExome BeadChip v1.0 (Illumina Inc., San Diego, CA, USA)

Genotyping using the HumanExome BeadChip identified 42 sequence variants significantly associated with metastasis in the total patient cohort (Supplementary Table 1). LD values (Lewontin’s D′ and r2) between the 42 SNPs were calculated and the genotype frequencies for each polymorphism in both metastatic and non-metastatic PCa were analyzed using a logistic regression model (Figure 1). Of the 42 polymorphisms significantly associated with PCa the five highest ranked were, rs2241714, rs143790069, rs72821581, rs75992542 and rs56350726. The minor allele of the rs2241714 polymorphism was positively correlated with mPCa; ORs and significance levels for all five SNPs are shown in Table 3.

**Table 3:** Top five SNPs of the logistic regression analysis of exome array to associate with metastatic PCa

SNPID	Chromosome	Alleles	Gene	Minor Allele Frequency		OR (95% CI)	*p*-value
				Class 1	Class 2		
rs72821581	6	T>C	TMEM14B	0.08537	0.01356	0.1354 (0.05492–0.3336)	1.39E-05
rs75992542	9	A>G	RABGAP1	0.1098	0.02453	0.2027 (0.09368–0.4385)	5.04E-05
rs56350726	9	T>A	SLC28A3	0.2195	0.08133	0.3256 (0.1884–0.5627)	5.83E-05
rs143790069	15	G>A	THBS1	0.1098	0.01778	0.1509 (0.068–0.3347)	3.29E-06
rs2241714	19	C>T	B9D2	0.2125	0.4979	3.672 (2.123–6.349)	3.25E-06

**Figure 2:**
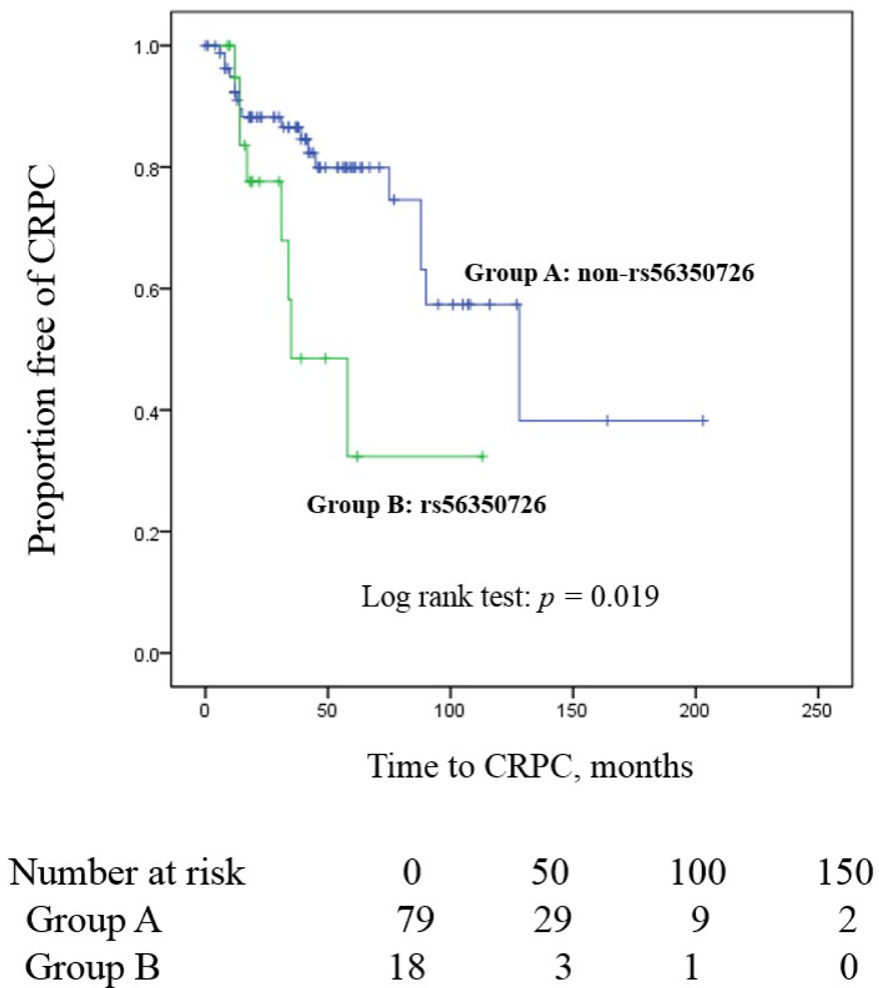
Kaplan-Meier curve illustrating the difference in time to CRPC between patients carrying the variant allele of rs56350726 compared to those that do not; Group A: non-rs56350726, Group B: rs56350726

As for progression free of CRPC survival, we found a significant difference between patients with non- rs56350726 and patients with rs56350726 (5-year CRPC free survival rate: 79.9% vs 32.3%; log-rank test: *p* = 0.019; Figure 2). No statistically significant association with CRPC-free survival was observed for rs2241714, rs143790069, rs72821581 or rs75992542 by analysis of data from patients treated with hormone therapy under the co-dominant model (*p* values for the Log-rank test were 0.074, 0.344, 0.452, and 0.307, respectively). Patients with visceral metastasis was observed 10 of 41 patients. According to visceral metastasis, we were not observed rapid progression to CRPC compared with only bony metastasis (Log Rank *p* = 0.057).

The Cox proportional hazards model showed an association between certain variables (Age, PSA and genetic genotyping) and progression to CRPC (Hazards ration [HR]: 0.959, *p* = 0.05; HR: 1.001, *p* < 0.001; HR: 2.734, *p* = 0.015, respectively) (Table 4).

**Table 4:** Univariate and multivariate analyses for the predictor of progression to CRPC using genetic analysis

	Univariate		Multivariate	
	HR (95% CI)	*p*-value	HR (95% CI)	*p*-value
Age, yr	0.959 (0.919–1.000)	0.050	1.016 (0.951–1.084)	0.644
PSA	1.001 (1.001–1.002)	< 0.001	1.000 (0.998–1.001)	0.815
Biopsy Gleason score				
6	ref		ref	
7	0.384 (0.024–6.177)	0.500	0.457 (0.028–7.531)	0.584
8	6.483 (0.870–48.299)	0.068	7.670 (0.932–63.088)	0.058
rs2241714	0.516 (0.246–1.084)	0.081	1.702 (0.572–5.066)	0.339
rs143790069	1.590 (0.600–4.214)	0.351	0.666 (0.163–2.730)	0.573
rs72821581	1.573 (0.475–5.214)	0.459	1.170 (0.283–4.837)	0.828
rs75992542	1.593 (0.644–3.945)	0.314	2.825 (0.685–11.656)	0.151
rs56350726	2.734 (1.215–6.155)	0.015	4.172 (1.223–14.239)	0.023

Multivariate Cox model including Age, PSA, biopsy Gleason score, rs2241714, rs143790069, rs72821581, rs75992542, rs56350726

Multivariable Cox proportional hazards regression analysis including age, PSA, biopsy Gleason score and genetic genotyping showed that rs56350726 were independent predictors of risk for progression to CRPC among patients who were treated with hormone therapy [Hazard ratio (HR): 4.172 (1.223–14.239), *p* = 0.023]. (Table 4).

## DISCUSSION

This study assessed potential genetic biomarkers for mPCa and investigated the utility of these markers for outcome prediction among patients who were treated with hormone therapy. Logistic regression analysis suggested that the top five SNPs significantly associated with risk for mPCa (compared to non-metastatic PCa) were rs2241714, rs143790069, rs72821581, rs75992542 and rs56350726. In addition, we found that rs56350726 was associated with progression to CRPC among mPCa patients treated with hormone therapy.

Two (rs75992542, rs56350726) of the top five SNPs associated with mPCa are located on chromosome 9, while the other three, rs2241714, rs143790069 and rs7282158, are located on chromosomes 19, 15 and 6, respectively. Three of the SNPs, rs2241714, rs7282158 and rs56350726, are intronic variants and the other two, rs143790069, rs75992542, encode missense changes within exons. The genes in which each of the SNPs are located are as follows; rs2241714, B9D2 and TMEM91; rs143790069, THBS1; rs7282158, TMEM14B; rs75992542, RABGAP1; and rs56350726, LOC105376116 and SLC28A3, respectively. rs2241714 is located in the promotor of the TGFB1 gene and it is associated with lung cancer [7]. rs143790069, rs72821581 and rs75992542 has not been kwon the function of these SNPs.

It is thought that, when the number of altered cells in a tumor increases, PCa progresses and metastasis occurs, in a process analogous to Darwinian evolution [8]. This evolutionary theory suggests that malignant transformation is a process with multistage mutations in oncogenes and tumor suppressor genes and also provides an opportunity for changes in cancer cells leading to them becoming more similar to non-malignant cells. Increasing clone heterogeneity is associated with the capacity to resist changes in the tumor microenvironment. Gundem et al. reported that this evolutionary process correlates with tumor metastasis [9]. The authors suggest two mechanisms for metastasis based on a whole genome genotyping study. The first model involves cross-metastatic seeding, which is the transfer of multiple cancer clones to distinct metastatic sites. This was demonstrated to be a therapy-induced event in a patient with two distinct metastatic lesions, one of which was derived from the original clone and the other from a clone localized to a distant metastasis [10]. The second mechanism is the de novo monoclonal seeding of daughter metastases.

Several prognostic factors have been reported in advanced PCa, including initial hemoglobin, serum albumin, erythrocyte sedimentation rate, lactate dehydrogenase, alkaline phosphatase, performance status and body weight after PSA relapse of PCa [11–14]. Some factors are associated not only with features of the cancer, but also with the general condition of the patient. Hence, the prognostic effects of these factors may not be directly associated with characteristics of the cancer. Although PSA levels are a useful tool for determining the effects of hormone therapy in advanced PCa, the prognostic importance of PSA indices after hormone therapy is debatable [15]. Moreover, few studies report whether PSA indices accurately predict progression to CRPC. Genetic variants have the potential to provide useful prognostic information in PCa. Gleason score and PSA are the important clinical predictive value for prognosis in patients with PCa. However, PCa is also crucially influenced from genetic background such as genetic variation. Patients with genetic variation are shown more easily manifestation to PCa.

The differences we observed in time to progression to CRPC are likely to result from gene–gene interactions of potential clinical importance. This finding requires further validation; however, the results supports our hypothesis that genetic variants associated with mPCa can also influence progression to CRPC after ADT, and are consistent with the intracrine model of resistance to ADT.

SLC28A3, encoding human concentrative nucleoside transporter-3 (hCNT3), is a key factor mediating the transport of purine and pyrimidine nucleosides [16, 17] and synthetic anticancer nucleosides, and is associated with drug toxicity [18, 19]. Moreover, SLC28A3 predicts clinical outcome among patients receiving gemcitabine plus paclitaxel for treatment of metastatic breast cancer [20]. Interestingly, we found that a variant in SLC28A3 is associated with metastasis and progression to CRPC in mPCa. We hypothesize that SLC28A3 could play an important role in transporting ADT, particularly in mPCa. This hypothesis will require further study. Another possibility is that SLC28A3 could play a role in androgen synthetic pathways. Errasti-Murugarren et al. reported that a splice variant of the SLC28A3 gene, encodes a hCNT3 protein localized to the endoplasmic reticulum (ER) [21]. The authors suggest that the variant protein could be involved in the salvage of intracellular nucleosides from the lumen of the ER to the cytoplasm.

STAMPEDE and Chaarted trials were shown higher tumor burden had better be treated by early chemotherapy [22, 23]. And previous studies suggested cross resistance to ADT with genetic analysis. PCa has the heterogeneity and it lead to resistance to ADT [24, 25]. Therefore, genetic variation which related with metastasis can be associated with the progression to CRPC.

Novel techniques have provided opportunities to investigate complexity at the level of a single cell. These have the potential to provide new information fundamentally related to heterogeneity, carcinogenesis and the metastatic process in PCa. Our results suggest a role for genetic variants in promoting PCa metastasis and progression to CRPC and provide the possibility of novel biomarkers, not only for the early detection of mPCa, but also for the assessment of PCa responses to hormone therapy. In addition, our findings present an opportunity to develop novel therapeutic targets to optimize a personalized approach for patients with mPCa.

We recognize that this study has some limitations. First, the discrepancy between the numbers in each group among the total cohort; however, all men included in this study were from a homogenous racial population. Second, ethnic differences (genetic or environmental factors) between Asians and Caucasians may translate to differences in the cancer microenvironment, which could present as ethnic differences in clinical outcomes, such as time to progression to CRPC. Furthermore, PCa is a hormone-dependent tumor, similar in that regard to breast cancer, and several investigators have suggested that ethnic variations in the serum levels of hormones, such as testosterone, may influence PCa risks and prognoses in different racial groups [26, 27]. Song et al. reported that differences in the hormone environment may be a key factor in the differences observed in the aggressiveness of PCa among Korean, compared to Western, men [28]. These results demonstrate that ethnic differences may influence innate differences in PCa, due to genetic variations; therefore, our results may not be applicable to other ethnic populations. Another limitation was the discrepancy in numbers in the different treatment groups. We did not found difference of clinical course between patients with ADT and orchiectomy. However, the number of patients with orchiectomy were relative small size in this study. We could not analyze impact of the type and duration of ADT to the progression of disease. Because some patients were treated with intermittent ADT and some patients were changed type of ADT. Our cohort included a relatively large number in the group receiving ADT compared to those that did not. Hence, our data represent a relatively small number of patients treated with hormone therapy. We did not the whole genome analysis, but we used commercial Kit which including exome and some sequencing including intron. These sequencing were used as an available tool for sequencing analysis instead of whole sequencing analysis.

Despite these limitations, a SNP associated with both metastatic PCa and progression to CRPC was identified. Our finding should assist both patients and clinicians in predicting outcomes of treatment with hormone therapy for metastatic PCa. Our results have the potential to lead to the development of a novel and clinically adaptable biomarker. For this to occur, a large scale, multiracial study is required to validate our results.

## MATERIALS AND METHODS

### Study population

With the approval of the institutional review board of Seoul National University Bundang Hospital, 1005 PCa patients were enrolled to this study from November 2003 to July 2013. Five patients, for whom no samples were available, were excluded. Therefore, genetic variation associated with metastatic PCa was evaluated in 1000 patients. The 1000 patients were stratified into two groups according to presence or absence of metastasis as determined using CT or MRI. All patients were diagnosed with adenocarcinoma of the prostate by biopsy of 12 or more cores. We assessed complete records of serum PSA, clinical stage, biopsy Gleason Score of all prostate biopsy cores, and pathologic outcomes if available. In addition, 110 of the study 1000 patients were treated with hormone therapy. Among these 110 patients, time to progression was defined as the duration from the date of ADT initiation to progression to CRPC. CRPC was defined as three consecutive increments in PSA resulting in two 50% increases over the nadir despite consecutive ADT, or progression, or the appearance of two lesions on a bone scan, or of soft tissue lesions on MRI or CT scan [29]. Metastatic PCa in this study defined M1 at the time of diagnosis.

### Pathological evaluation

All patients underwent transrectal ultrasound (TRUS)-guided biopsies of 12 or more cores using an automatic firing mechanism. Systematic biopsies included samples close to the bilateral apex, mid zone and base gland, with six or more biopsies per lobe. If any suspicious lesions were observed, additional core biopsies were performed. A single genitourinary pathologist (G.C.) performed the pathologic analysis. The 2005 International Society of Urological Pathology (ISUP) guidelines were updated to adopt a modified Gleason score (GS) system to reflect clinical practice and improve understanding prostate cancer pathophysiology [30]. According to the update, patients were reviewed from 2006 at our institution. And patient data from 2003 to 2005 was reviewed according to 2005 ISUP.

### Genotyping and quality control

Blood specimens were collected from all patients prospectively into tubes containing sodium EDTA and the QIAamp Blood Extraction kit (Qiagen, Seoul, Korea) was used for DNA extraction. These samples were analyzed using the HumanExome BeadChip 12v1-1 (Illumina, Inc., San Diego, CA). This chip includes 242,901 markers focusing on protein-altering variants. Information about the exome array design, marker selection strategies and single nucleotide polymorphism (SNP) content is available at the exome array design webpage (http://genome.sph.umich.edu/wiki/Exome_Chip_Design).

Genotypes were called using Illumina’s GenTrain version 2.0 clustering algorithm within GenomeStudio software (V2011.1). Cluster boundaries were chosen using Illumina’s standard cluster file. SNPs with call rates of < 0.99 were visually inspected. The majority (242,186 of 242,901; 99.71%) of genotyped SNPs with minor allele frequencies (MAFs) > 0.002, were successfully mapped with a call rate of > 95%. The average call rate was 99.98%. In total, 999 of 1,000 (99.9%) individuals were successfully genotyped (call rate > 98%). As a quality control measure, concordance was determined for the 242,186 SNP genotypes for 104 blind duplicate sample pairs and the results indicated 99.998% agreement. We excluded one individual per pair of six known twin pairs and six unexplained apparent duplicates. Principal components analysis (PCA) was conducted twice; first, with HapMap samples excluded to facilitate identification of population outliers, and second, with HapMap samples included to support the interpretation of the outlier results. To reduce the risk of artifactual bias caused by familial relatedness, principal components (PCs) were computed using estimated SNPs from a subset of 7,304 unrelated individuals. Close relatives was defined as individuals for whom the estimated genome wide identical-by-descent (IBD) proportion of alleles shared was > 0.10. Estimated IBD was determined using PLINK’s ‘‘-genome’’ option38, and PCA was conducted using SMARTPCA37 on a linkage-disequilibrium-pruned set of 22,464 autosomal SNPs. After removing large scale high linkage disequilibrium (LD) regions, SNPs with Hardy-Weinberg equilibrium (HWE) *p* values of < 10^–4^ and SNPs with MAFs < 0.01, LD pruning was conducted using the PLINK option, ‘‘-indep-pairwise 50 5 0.2’’. The first ten PCs were inspected and 12 population outliers were identified. Nine of them had self-reported non-Finnish ancestry and these 12 individuals were excluded from subsequent analysis.

### SNP analysis

The SNP genotype frequencies of the total cohort were evaluated using the chi-square test, and all were consistent with HWE (*P* > 0.05). Odds ratios (ORs) were calculated as estimates of the relative risk of metastatic PCa associated with each SNP genotype. Data were analyzed using a logistic regression analysis to avoid confounding factors, and then relationships between genotypes and haplotypes were determined. Lewontin’s D’ (|D’|) and the LD coefficient r^2^ were calculated to estimate LD between all biallelic pairs of loci [31]. Haplotypes were predicted from the genotyped SNPs with the PHASE algorithm ver. 2.0 [32], using SAS version 9.1 (SAS Inc., Cary, NC, USA).

### Statistical analysis

A total of 1000 PCa patients were stratified according to metastatic status. When comparing patients with and without metastasis, we assessed differences in the clinical profiles of patients using the Student t test or the chi-squared test. The clinical characteristics of 110 of the 1000 PCa patients who received ADT were analyzed using the Student t test and chi-square test.

We assumed a co-dominant effect of the variant (V) wild-type (W) alleles in one analysis model, where genotypes W/W, W/V and V/V were coded as 0, 1 and 2, respectively. If a dominant effect was assumed, genotype W/W was coded as 0, whereas genotypes W/V and V/V were both coded as 1. Accordingly, the combined genotypes W/W and W/V were scored as 0, and V/V as 1 in a model assuming a recessive effect [33].

The CRPC-free survival rates of patients, according to SNPs associated with metastatic PCa, were calculated and compared using the Kaplan-Meier (K-M) log-rank test. Univariable and multivariable Cox proportional hazard models were used to analyze predictive factors associated with progression to CRPC. SPSS 22.0 for Windows (IBM^®^ SPSS^®^ version 22.0, IBM, Armonk, New York, USA) was used for statistical analyses. We considered 2-tailed *p* values of < 0.05 as statistically significant for all analyses.

## CONCLUSIONS

The top five SNPs associated with metastatic PCa were rs2241714, rs143790069, rs72821581, rs75992542 and rs56350726. Moreover, one of these SNPs (rs56350726 in SLC28A3) was shown to be significantly associated with differences in progression to CRPC, among patients treated with hormone therapy. A large scale, multi-ethnic study is required to validate our results, which have the potential to predict progression to CRPC among patients diagnosed with mPCa.

## SUPPLEMENTARY MATERIALS TABLE


