# Correction: A genetic variant in SLC28A3, rs56350726, is associated with progression to castration-resistant prostate cancer in a Korean population with metastatic prostate cancer

**DOI:** 10.18632/oncotarget.25970

**Published:** 2018-08-07

**Authors:** Jung Ki Jo, Jong Jin Oh, Yong Tae Kim, Hong Sang Moon, Hong Yong Choi, Seunghyun Park, Jin-Nyoung Ho, Sungroh Yoon, Hae Young Park, Seok-Soo Byun

**Affiliations:** ^1^ Department of Urology, Hanyang University Hospital, Seoul, Korea; ^2^ Department of Urology, Seoul National University College of Medicine, Seoul National University Bundang Hospital, Seongnam, Korea; ^3^ Department of Urology, Hanyang University Guri Hospital, Guri-si, Korea; ^4^ Department of Electrical and Computer Engineering, Seoul National University, Seoul, Korea; ^5^ Biomedical Research Institute, Seoul National University Bundang Hospital, Seongnam, Korea

**This article has been corrected:** The correct author name is given below:

**Jung Ki Jo**

Original article: Oncotarget. 2017; 8:96893-96902. https://doi.org/10.18632/oncotarget.18298

