# Supplementary material for: A genetic variant in SLC28A3, rs56350726, is associated with progression to castration-resistant prostate cancer in a Korean population with metastatic prostate cancer
Source: Oncotarget. 2017 May 30;8(57):96893–902. doi: 10.18632/oncotarget.18298 (PMC5722531; doi:10.18632/oncotarget.18298)
Supplement: Supplementary file 1 [file oncotarget-08-96893-s001.pdf]

A genetic variant in SLC28A3, rs56350726, is associated with progression to castration-resistant prostate cancer in a Korean population with metastatic prostate cancer

Supplementary Materials

MATERIALS AND METHODS

Abbreviations

Castration-resistant prostate cancer: CRPC; Androgen-deprivation therapy: ADT; Prostate cancer: PCa; Metastatic prostate cancer: mPCa; Single nucleotide polymorphism: SNP; Next-generation sequencing: NGS; Computed tomography: CT; Magnetic Resonance Imaging: MRI; Prostate Specific Antigen: PSA; Trans-Rectal Ultrasound: TRUS; Deoxyribonucleic acid: DNA; Minor allele frequency: MAF; Principal components analysis: PCA; Principal component: PC; Identical-by-descent: IBD; Linkage disequilibrium: LD; Hardy-Weinberg equilibrium: HWE; Odds ratio: OR; Kaplan-Meier: K-M; Body mass index: BMI

Supplementary Table 1: Logistic regression analysis of SNPs with the risk of metastatic PCa

SNPID	Chromosome	Alleles	Gene	Minor Allele Frequency		OR (95% CI)	*p*-value
				Class 1	Class 2		
rs2241714	19	C>T	B9D2	0.2125	0.4979	3.672 (2.123–6.349)	0.00000325
rs143790069	15	G>A	THBS1	0.1098	0.01778	0.1509 (0.068–0.3347)	0.00000329
rs72821581	6	T>C	TMEM14B	0.08537	0.01356	0.1354 (0.05492–0.3336)	0.0000139
rs75992542	9	A>G	RABGAP1	0.1098	0.02453	0.2027 (0.09368–0.4385)	0.0000504
rs56350726	9	T>A	SLC28A3	0.2195	0.08133	0.3256 (0.1884–0.5627)	0.0000583
rs10868138	9	T>C	SLC28A3	0.2073	0.07873	0.3287 (0.1866–0.5791)	0.0001178
rs1555300	20	A>G	RIMS4	0.6463	0.4248	0.413 (0.2609–0.654)	0.0001627
rs4819522	22	C>T	TBX1	0.2317	0.09645	0.3588 (0.2095–0.6144)	0.0001876
rs795791	15	A>C	TEX9,MNS1	0.1951	0.4062	2.917 (1.66–5.123)	0.0001961
rs12280680	11	G>A	CREB3L1	0.1951	0.07612	0.3243 (0.1785–0.589)	0.0002173
rs3785522	17	G>A	TBCD	0.1463	0.04854	0.2728 (0.137–0.543)	0.0002174
rs146571920	15	A>C	CASC5	0.07317	0.01408	0.169 (0.06557–0.4356)	0.0002328
rs146933458	2	C>T	KIF5C	0.1585	0.05683	0.2762 (0.1389–0.5492)	0.0002437
rs10437653	11	A>C	.	0.1951	0.07716	0.3284 (0.1807–0.5967)	0.0002577
rs1190788	6	G>A	AKAP7	0.06098	0.009906	0.1455 (0.05145–0.4117)	0.0002803
rs7674577	4	G>A	.	0.09756	0.2891	3.936 (1.867–8.298)	0.0003177
rs146536550	1	C>G	ZZZ3	0.07317	0.01199	0.1995 (0.08218–0.4842)	0.0003669
rs1935881	1	A>G	.	0.2439	0.1077	0.3953 (0.2369–0.6596)	0.0003816
rs17410015	1	T>C	.	0.07317	0.01512	0.1819 (0.07094–0.4664)	0.0003888
rs9376092	6	C>A	.	0.5732	0.37	0.4491 (0.2884–0.6995)	0.0003985
rs9402686	6	G>A	.	0.5732	0.3707	0.451 (0.2897–0.702)	0.0004211
rs4895441	6	A>G	.	0.5732	0.3707	0.451 (0.2897–0.702)	0.0004211
rs17747633	15	A>G	CASC5,CASC5	0.1463	0.04901	0.3191 (0.1691–0.6025)	0.0004266
rs9373124	6	T>C	.	0.5732	0.3711	0.4515 (0.29–0.7029)	0.0004308
rs586024	6	A>G	GPR116	0.06098	0.01095	0.1612 (0.05752–0.4518)	0.0005181
rs10152546	15	T>A	C15orf57	0.1463	0.05005	0.3246 (0.1719–0.613)	0.0005228
rs7784776	7	G>A	.	0.4268	0.2492	0.4564 (0.2922–0.7128)	0.0005634
rs11253684	10	G>A	.	0.1098	0.03233	0.263 (0.123–0.562)	0.0005672
rs147272153	19	G>A	PHLDB3	0.07317	0.0146	0.2036 (0.08216–0.5046)	0.0005885
rs690347	15	G>T	ZFYVE19	0.1707	0.06569	0.3468 (0.1894–0.6351)	0.0006026
rs139626065	5	C>T	GPR151	0.06098	0.01147	0.1691 (0.06056–0.4719)	0.0006888
rs150152777	20	T>G	C20orf152	0.06098	0.01147	0.1691 (0.06056–0.4719)	0.0006888
rs41273541	1	T>C	OR6N2	0.3293	0.1794	0.429 (0.2622–0.702)	0.0007558
rs3739040	2	C>T	LRRFIP1	0.2073	0.0902	0.3873 (0.2224–0.6743)	0.0007993
rs3739039	2	A>G	LRRFIP1	0.2073	0.0902	0.3873 (0.2224–0.6743)	0.0007993
rs74530614	19	C>A	FFAR1	0.09756	0.02815	0.2461 (0.1084–0.5587)	0.0008027
rs7460258	8	C>T	.	0.4634	0.2865	0.4641 (0.2962–0.7272)	0.0008059
rs17167553	7	G>T	LRGUK	0.5	0.3227	0.4581 (0.2895–0.7248)	0.0008547
rs2229889	20	C>T	RRBP1	0.07317	0.01566	0.2163 (0.08777–0.5329)	0.0008757
rs9606756	22	A>G	TCN2,TCN2	0.06098	0.01199	0.1769 (0.06362–0.492)	0.0009032
